# Targeting the Enhanced Sensitivity of Radiotherapy in Cancer: Mechanisms, Applications, and Challenges

**DOI:** 10.1002/mco2.70202

**Published:** 2025-05-15

**Authors:** Yuanyuan Zhao, Fangqin Tan, Jiajia Zhao, Shuchang Zhou, Yao Luo, Chen Gong

**Affiliations:** ^1^ Department of Oncology Department of Radiology Institute of Organ Transplantation Tongji Hospital Tongji Medical College Huazhong University of Science and Technology Wuhan China; ^2^ Key Laboratory of Organ Transplantation Ministry of Education NHC Key Laboratory of Organ Transplantation Key Laboratory of Organ Transplantation Chinese Academy of Medical Sciences Organ Transplantation Clinical Medical Research Center of Hubei Province Wuhan Wuhan China; ^3^ Department of Stomatology Union Hospital Tongji Medical College Huazhong University of Science and Technology Wuhan China; ^4^ Department of Laboratory Medicine Sichuan Clinical Research Center for Laboratory Medicine West China Hospital Sichuan University Chengdu China

**Keywords:** combination therapy, radiosensitization, radiosensitizers, radiotherapy

## Abstract

Cancer is a major public health, societal, and economic challenge worldwide. According to *Global Cancer Statistics 2022*, it is estimated that by 2050, there will be 35 million new cancer cases globally. Although patient survival rates have improved through various therapeutic approaches, including surgery, chemotherapy, and radiotherapy, treatment efficacy remains limited once tumor metastasis occurs. Among various cancer treatment strategies, radiotherapy plays a crucial role. Along with surgery and chemotherapy, radiotherapy is a cost‐effective single‐modality treatment, accounting for approximately 5% of total cancer care costs. The use of radiosensitizing agents such as histone deacetylase inhibitors, 2‐deoxy‐d‐glucose, enterolactone, and squalene epoxidase can enhance radiotherapy effectiveness. Recent radiosensitization methods involve physical stimuli and chemical radiosensitizers. However, improving their efficacy, durability, and overcoming radioresistance remain significant challenges. This review first introduces current applications of radiotherapy in cancer treatment, the molecular mechanisms underlying its anticancer effects, and its side effects. Second, it discusses the main types of radiosensitizers, their latest applications, and recent challenges in cancer treatment. Finally, it emphasizes on clinical trials of radiosensitizing agents and explores potential biomarkers for radiotherapy response in cancer. Multifunctional nanoparticles have shown greater clinical applicability than single‐functional nanoparticles. Future research will focus on enhancing the drug‐carrying capacity of nanomaterials to further improve radiotherapy outcomes.

## Graphical abstract

In this review, we first introduced the current application scenarios for radiotherapy in tumor treatment, the molecular mechanisms underlying anticancer effects of radiotherapy in treatment of cancer, and the side‐effects of radiotherapy. Second, we pay attention to discuss the main types of radiosensitizers and their latest applications and challenges in the tumor treatment. Furthermore, we also focus on the clinical trials of radiosensitizing agents/methods and predict biomarkers to radiotherapy in cancer

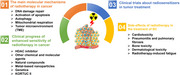

## 1 Introduction

Cancer is a major global public health, societal, and economic concern. According to *Global Cancer Statistics 2022*, cancer accounts for three in 10 global premature deaths from noncommunicable diseases (30.3% in individuals aged 30–69 years), making it one of the three leading causes of death in this age group across 177 of 183 countries [1]. By 2050, cancer incidence is projected to reach 35 million new cases worldwide [1]. For instance, in China, female breast cancer is the most commonly diagnosed cancer across all levels of the Human Development Index. In the United States, breast cancer accounts for 32% of newly diagnosed tumors in women in 2024 [2]. Over the past decade (2012–2019), incidence rates have risen significantly among younger women (≤50 years) at 1.1% per year, compared with 0.5% per year in those aged ≥50 years [2]. This trend is expected to continue, leading to higher mortality rates.

Despite advancements in cancer therapies, including surgery, chemotherapy, and radiotherapy, treatment efficacy remains limited once tumor metastasis occurs [3]. However, increasing knowledge of tumor molecular characteristics, including the tumor microenvironment (TME) and immune system interactions, has provided new insights into tumor genotyping, diagnosis, and treatment strategies [3]. Breast cancer, for example, is recognized as a heterogeneous disease with clinically distinct subtypes based on estrogen receptor (ER), progesterone receptor, and human epidermal growth factor receptor 2 (HER2) expression [4]. Additionally, Ki‐67 serves as a valuable biomarker for luminal breast cancer subtypes due to its strong association with tumor cell proliferation [5]. Understanding molecular characteristics is essential for diagnosing breast cancer and other malignancies, selecting therapeutic regimens, and assessing prognosis.

Among cancer treatment strategies, radiotherapy plays a vital role [6]. It employs high doses of ionizing radiation to destroy cancer cells and prevent tumor recurrence. Over 70% of lung cancer patients require radiotherapy at some stage of disease progression [7]. Alongside surgery and chemotherapy, radiotherapy remains a cost‐effective single‐modality treatment, constituting approximately 5% of total cancer care costs [8]. Combining surgery with radiotherapy is a common approach to improving local breast cancer control and achieving favorable survival outcomes [9]. Radiotherapy is also a standard neoadjuvant treatment for intermediate and locally advanced rectal cancer [10]. The landmark *American College of Surgeons Oncology Group (ACOSOG) Z0011* trial demonstrated no significant difference in outcomes between lumpectomy and breast radiation [11]. Recent large‐scale clinical trials have revealed that adding regional nodal irradiation (IR) to whole‐breast or chest wall IR results in a greater absolute reduction in distant metastases than in locoregional recurrences [12, 13]. Nevertheless, metastatic cancer remains challenging to cure with current therapies, carrying a poor prognosis [14]. Treatment approaches for metastasis include radiotherapy, chemotherapy, and surgery, with intervention strategies determined by metastasis extent and location. For example, in breast cancer, primary tumors and metastatic lesions can be managed using surgery, local ablative therapies, and radiotherapy combined with systemic treatment. Treatment decisions depend on disease stage, pathological characteristics, and molecular profiles. Clinical data suggest that patients with oligometastatic disease (defined as ≤5 metastatic lesions) who undergo radiotherapy or stereotactic ablative radiotherapy may experience improved progression‐free survival and overall survival compared with standard care [15, 16]. Recent advancements in imaging techniques, computerized treatment planning, radiation therapy machines, and radiobiology have significantly enhanced radiotherapy outcomes [17]. Efforts to overcome tumor radioresistance have driven extensive research into effective radiosensitization strategies [18]. Radiosensitization methods typically involve physical stimuli (e.g., heat, light, sound) [19] and chemical radiosensitizers (e.g., small molecules, nanomaterials, macromolecules) [17].

However, challenges remain in improving the efficacy, durability, and overcoming resistance to radiosensitizers. The application and suitability of radiotherapy in breast cancer treatment remain under debate. Additionally, developing novel radiotherapy biomarkers to predict therapeutic response and prognosis could contribute to more effective clinical strategies. With the rapid advancement of molecular biology, research into potential molecular targets for radiosensitization has become a critical focus.

Based on these backgrounds, this review examines the latest applications of radiotherapy in cancer treatment, the molecular mechanisms underlying its anticancer effects, and its associated side effects. It further explores the primary types of radiosensitizers, their clinical applications, and challenges in cancer treatment. Finally, it discusses ongoing clinical trials of radiosensitizing agents and evaluates potential biomarkers for radiotherapy response.

## 2 The Main Molecular Mechanisms of the Sensitivity and Resistance of Radiotherapy in Cancer

In recent decades, advances in understanding the molecular mechanisms and signaling pathways involved in radiosensitization and radiotherapy resistance have facilitated the clinical application and optimization of radiotherapy in cancer treatment. DNA damage repair (DDR), cellular apoptosis activation, autophagy, and mitochondrial respiration are the four key pathways regulating radiotherapy sensitivity and resistance.

### 2.1 DNA Damage Repair

**FIGURE 1:**
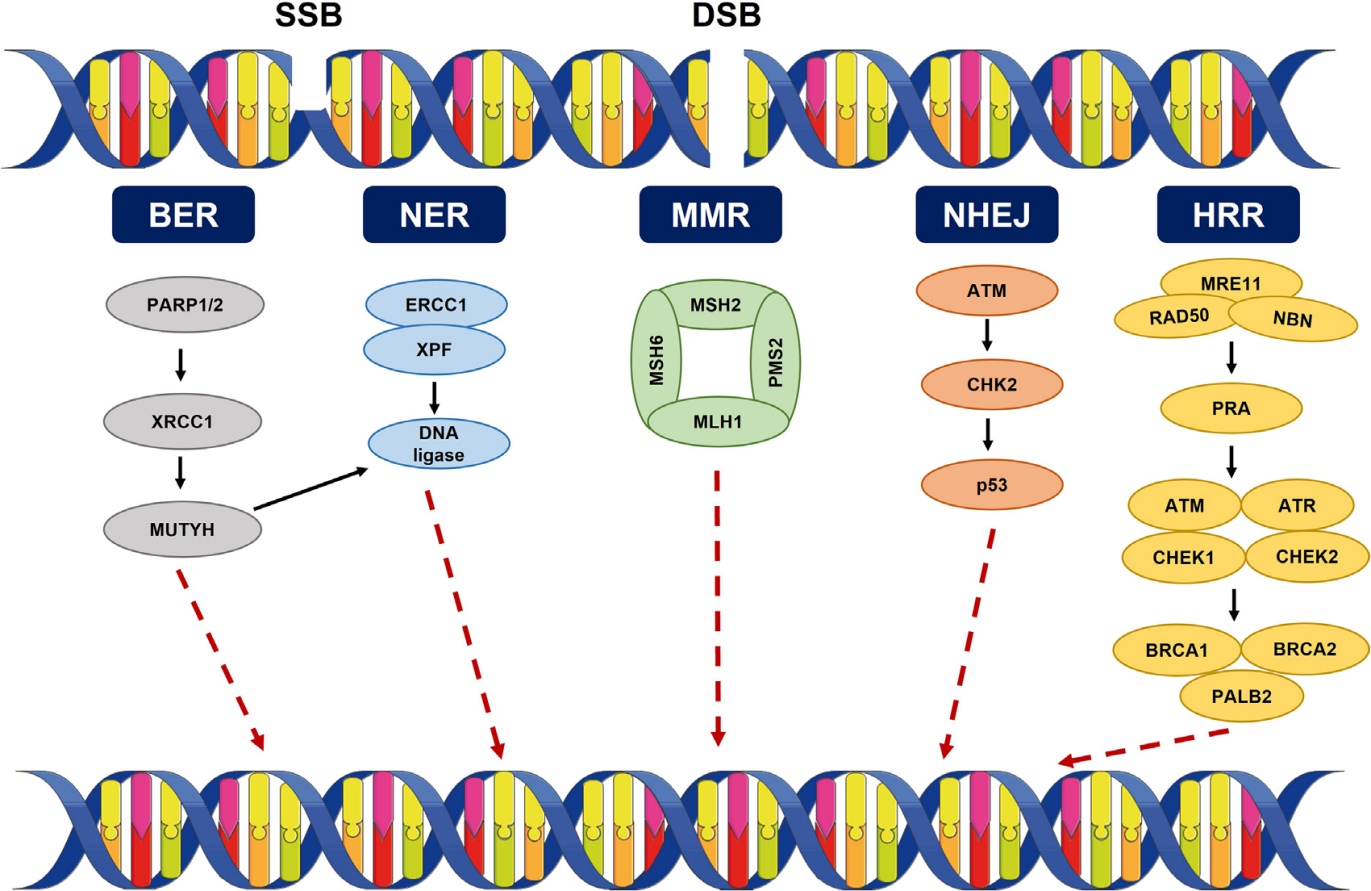
DNA damage repair. SSB is repaired by the BER, NER, and MMR machineries. DSB is repaired by HRR and NHEJ. *Abbreviations*: SSB, single strand break; DSB, double strands break; MMR, mismatch repair; NER, nucleotide excision repair; BER, base excision repair; HRR, homologous recombination repair; NHEJ, nonhomologous end joining.

A primary mechanism by which ionizing radiation kills cancer cells is through the induction of DNA damage. Radiation generates abundant free radicals, primarily reactive oxygen species (ROS), which cause DNA modifications and damage [20]. The types of DNA damage include base lesions, single‐strand breaks (SSBs), double‐strand breaks (DSBs), and sugar damage. Several mechanisms and pathways involved in DDR influence radiotherapy sensitivity (Figure 1).

During the synthesis (S) and gap 2 (G2) phases of the cell cycle, homologous recombination repair (HRR) plays a crucial role in DNA repair. HRR requires the coordinated action of multiple enzymes, primarily nuclease MRE11, RAD50, and nibrin, which together form the MRN complex [21]. The MRN complex initiates 5′ to 3′ DNA end resection, which is protected from degradation by replication protein A (RPA). Subsequently, MRN and RPA activate kinases such as ATM, ATR, CHEK1, and CHEK2, which recruit BRCA1, PALB2, and BRCA2 to facilitate the exchange of RPA with recombinase RAD51 [22, 23]. RAD51 is a key HRR protein that forms a nucleoprotein filament, while its paralog, XRCC3, further enhances the process [24]. Ultimately, RAD51 facilitates the formation of Holliday junction intermediates, leading to complete DSB repair. Enhanced DDR mechanisms contribute to radiation resistance in tumor cells. For instance, selective inhibition of RAD51 expression has been shown to enhance the cytotoxic effects of radiation. Furthermore, molecular inhibitors targeting RPA subunits have demonstrated potential in improving radiotherapy response [25].

Beyond DSB repair, other DNA repair mechanisms, including base excision repair (BER), mismatch repair (MMR), and nucleotide excision repair (NER), can also be employed by cancer cells. The DNA repair protein XRCC1 is involved in nearly every step of BER via the short‐patch repair pathway [26]. Certain XRCC1 variants have been associated with an increased risk of breast cancer and adverse reactions to radiotherapy [27]. However, contradictory evidence suggests that XRCC1 overexpression correlates with improved cancer‐specific survival in bladder cancer patients [28].

Another key protein, poly(ADP‐ribose) polymerase (PARP), plays a vital role in DNA repair, replication, transcription, and posttranslational modification. PARP inhibitors have been shown to modulate responses to ionizing radiation in non‐small cell lung cancer (NSCLC) [29]. Recent studies have identified a new class of dual PARP‐1/proteasome inhibitors with significant effects on chemotherapy and radiotherapy outcomes [30, 31].

DDR is critical for maintaining genomic stability and integrity. Rapid tumor growth and abnormal blood vessel formation contribute to tumor hypoxia, which impairs radiation‐induced DNA damage fixation and reduces radiotherapy efficacy [32]. Additionally, recent studies indicate that radiotherapy induces PD‐L1 upregulation in cancer cells, reinforcing DDR pathways and ultimately enhancing tumor resistance [33, 34]. Tumors exploit their own repair genes, primarily utilizing HRR or nonhomologous end joining (NHEJ) pathways [35]. Deficiencies in DNA repair genes increase radiotherapy sensitivity, while overexpression of these genes has been associated with radiotherapy resistance.

### 2.2 Activation of Apoptosis

In addition to DNA damage, activation of apoptotic pathways is a crucial molecular mechanism influencing radiotherapy sensitivity. Apoptosis is a highly regulated form of cell death characterized by distinct morphological and molecular features [36]. Tumor cells inevitably undergo programmed cell death following exposure to ionizing radiation, a process known as type I programmed cell death or apoptosis.

The tumor suppressor protein TP53 (p53) plays a pivotal role in apoptosis. Inanami et al. [37] found that a low concentration of ECyd enhanced radiation‐induced apoptosis in a TP53‐independent manner in human gastric cancer cell lines MKN45 (TP53 wild‐type) and MKN28 (TP53‐mutated). Conversely, negative modulation of the TP53 signaling pathway, such as by CDK16, promoted radioresistance [38]. Typically, TP53 induces the expression of proapoptotic factors such as BAX and PUMA [39]. Activation of these signaling pathways leads to cytochrome c release, activation of caspase cascades, and ultimately, apoptosis.

Sphingolipid metabolites, including ceramide, sphingosine, and sphingosine‐1‐phosphate (S1P), play crucial roles in regulating apoptosis. Among these, S1P suppresses ceramide‐dependent apoptosis, whereas ceramide and sphingosine mediate cell death, cell cycle arrest, and senescence upon radiation and other anticancer treatments [40]. Notably, ceramide‐dependent apoptosis is both dose‐ and time‐dependent in response to radiotherapy [41, 42].

Recent studies indicate that the circadian clock pathway PER1–ATM:CHEK2 can trigger TP53‐mediated apoptosis following radiation exposure [43]. Nelson et al. [44] proposed potential therapeutic strategies targeting circadian pathways to enhance breast cancer treatment outcomes. Furthermore, the PI3K/Akt/mammalian target of rapamycin (mTOR) signaling pathway plays a significant role in apoptosis. Dadey et al. [45] reported that antibody targeting GRP78 enhanced radiotherapy efficacy in glioblastoma and NSCLC by inhibiting the PI3K/Akt/mTOR pathway and promoting apoptosis. Conversely, the PI3K/Akt/mTOR pathway also influences glucose metabolism and contributes to radioresistance by inhibiting the ubiquitination of rate‐limiting enzymes in the pentose phosphate pathway [46]. These findings underscore the critical role of apoptosis in determining tumor response to radiotherapy.

### 2.3 Autophagy

Autophagy was first identified as a distinct form of cell death in the 1970s. It is a highly conserved process that facilitates cellular degradation and recycling by transporting cytoplasmic components to lysosomes [47]. Studies have shown that autophagy plays dual roles in cancer, contributing to both tumor cell survival and cell death [48, 49].

In normal cells, autophagy prevents tumorigenesis by counteracting oncogenic stimuli [50]. and promoting oncogene‐induced senescence [51]. However, in cancer, multiple risk factors influence the functional consequences of autophagy [52]. Radiation‐induced stress elicits two primary autophagic responses in tumor cells: cytoprotective and cytotoxic functions [53]. Additionally, nonprotective and cytostatic autophagy have been identified as alternative responses [54].

During early tumorigenesis, autophagy acts as a tumor suppressor by eliminating damaged mitochondria and aberrant protein aggregates that produce ROS. Consequently, loss of autophagy‐related proteins and genes can promote cancer initiation. However, in established tumors, autophagy facilitates survival by helping cells withstand radiation‐induced stress [55].

Several key signaling pathways regulate tumor response to radiotherapy through autophagy. For instance, inhibition of the PI3K/Akt/mTORC1 pathway induces autophagy and enhances radiosensitivity in multiple tumor cell lines [56]. Additionally, autophagy agonists such as sirolimus and rapamycin increase the effectiveness of radiotherapy. Conversely, reduced expression of the Beclin‐1 (BECN1) gene enhances radioresistance. In breast cancer, BECN1 deficiency is frequently observed [57, 58]. BECN1, along with autophagy‐related genes (ATGs), is recruited by ULK1, leading to phagophore formation. Overexpression of BECN1 has been shown to suppress HER2‐enriched breast tumors [59], potentially through autophagic degradation of Notch1‐dependent tumorigenesis.

Approximately 20 ATGs actively participate in autophagy [60]. Some, such as ATG7, function as tumor suppressors, whereas oncogenic factors like Ras and signal transducer and activator of transcription 3 are frequently overexpressed in breast cancer and contribute to tumor progression [61, 62]. Forkhead Box O (FOXO) proteins also regulate cellular homeostasis and stemness. While FOXO1 is associated with poor prognosis [63], FOXO3 is linked to reduced metastatic events in luminal‐like breast cancer [64]. Loss of FOXO3 leads to diminished autophagic activity.

Autophagy plays a role not only in reducing drug sensitivity but also in tumor dormancy, hypoxia adaptation, chemoresistance, and the development of stem‐like phenotypes [49]. Because autophagy provides metabolic support, tumor cells can repair radiation‐induced damage, thereby promoting disease progression [65]. Moreover, autophagy aids tumor growth by facilitating distant colonization and extravasation of dormant cancer cells [66, 67]. The mTOR and AMP‐activated protein kinase pathways are essential regulators of this process [68]. Interestingly, autophagy enables cancer stem cell (CSC) survival under hypoxia in the TME [69]. CSCs contribute significantly to chemoresistance [70], with hypoxia‐inducible factor‐1 (HIF‐1) serving as a key regulator. Several pathways, including NANOG, SOX2, and SOX17, activate HIF‐1 expression [71, 72].

In radiotherapy, autophagy is stimulated to regulate cancer cell survival posttreatment [73]. Damage‐regulated autophagy modulator 1 (DRAM1) is involved in this process [74]. Additionally, autophagy‐mediated degradation of Ubqln1 plays a critical role in autophagosome maturation [75]. Several microRNAs (miRNAs), including miR‐26b and miR‐200C, modulate radiotherapy sensitivity by regulating DRAM1 and Ubqln1 expression, respectively [74, 76]. Taken together, these findings highlight the vital role of autophagy in cancer, influencing both radiotherapy sensitivity and resistance.

### 2.4 Mitochondrial Respiration

Mitochondria are essential for cellular energy production, playing a crucial role in maintaining cellular integrity, bioenergetics, metabolism, and signaling [77]. Recently, the influence of mitochondrial respiration on radiotherapy sensitivity has gained significant attention. Mitochondria contribute to genomic stability and cellular homeostasis during and after radiotherapy. Several reviews have summarized their role in enhancing radiotherapy efficacy across different cancer types [77, 78].

For instance, in breast cancer, Li et al. [79] observed that targeting Elongin B (ELOB) significantly enhanced radiotherapy efficacy in triple‐negative breast cancer (TNBC). ELOB is involved in transcription elongation and gene expression regulation. The study found that high ELOB expression was associated with poor prognosis in TNBC patients undergoing radiation therapy. Furthermore, ELOB inhibition impaired mitochondrial function and increased radiosensitivity in TNBC cell lines BT‐20, BT‐549, MDA‐MB‐468, and MDA‐MB‐436.

Additionally, arsenic trioxide (As₂O₃) has been shown to inhibit mitochondrial respiration, increase tumor oxygenation (pO₂) by reducing oxygen consumption, and ultimately enhance radiosensitivity in solid tumors. Diepart et al. [80] demonstrated in two murine models that irradiating tumors during periods of As₂O₃‐induced oxygenation led to a 2.2‐fold increase in radiosensitivity compared with control mice. These findings suggest that As₂O₃ could be a promising candidate for incorporation into IR protocols for solid tumors such as breast cancer.

Beyond As₂O₃, Crokart et al. [81] reported that glucocorticoids might enhance tumor radiosensitivity via a similar mechanism—by increasing tumor pO₂ through mitochondrial respiration inhibition. Specifically, when IR (25 Gy) was applied at *t*_max_, tumor radiosensitivity increased (regrowth delay was extended by a factor of 1.7) [81]. These findings highlight mitochondrial respiration inhibition as a promising strategy for enhancing radiosensitivity in future clinical applications.

### 2.5 Tumor Microenvironment

The TME represents the local environment surrounding a tumor. It consists of multiple components, including immune cells, fibroblasts, epithelial cells, extracellular matrix proteins, blood and lymphatic vessels, metabolites, chemokines, and cytokines [82]. Radiotherapy has been shown to modulate the TME, shifting it from an immunosuppressive to an immunostimulatory state [83].

A TME is classified as immunostimulatory when highly infiltrated by T lymphocytes, whereas an immunosuppressive TME lacks T‐cell infiltration. Radiotherapy induces this TME switch based on several factors [83].

1. Local production of chemokines, cytokines, and soluble factors
2. Modifications of tumor‐associated stroma and endothelium
3. Trafficking or modulation of immune cell subsets within the TME

Radiosensitivity is influenced by complex interactions between cancer cells and their immune TME [84]. For instance, the presence and activation status of dendritic cells (DCs), CD8⁺ T cells, and antitumor cytokines are key determinants of radiotherapy response [85, 86]. Furthermore, intratumoral immune responses following radiotherapy impact therapeutic outcomes. In the *KEYNOTE‐001* trial, patients who received radiation before immune checkpoint therapy exhibited significantly improved progression‐free survival, with median survival extending from 9.5 to 11.5 months [87]. The TME contains a diverse array of immune cells, each with distinct roles in regulating radiosensitivity [88]. Tumor‐associated macrophages (TAMs), myeloid‐derived suppressor cells (MDSCs), and CD4⁺ regulatory T cells are immunosuppressive and promote tumor progression. In contrast, immune cells such as DCs, CD8⁺ T cells, and natural killer cells exhibit antitumorigenic activity [89].

Radiation plays a critical role in TAM recruitment and phenotype modulation within the TME. Several factors regulate TAM recruitment. For example, in glioma models, radiation‐induced HIF‐1 expression promoted TAM infiltration, whereas blocking colony‐stimulating factor 1 reduced TAM accumulation in prostate cancer models [90, 91]. TAM‐mediated radioresistance is primarily driven by tumor necrosis factor signaling‐dependent upregulation of vascular endothelial growth factor [92]. Additionally, radiotherapy can induce an influx of MDSCs, leading to further polarization of the TME into an immunosuppressive state. Immune cells within the TME play crucial roles in mediating radiosensitivity [85, 86, 93]. Understanding how the TME affects immunotherapy responses may provide valuable insights for optimizing radiotherapy efficacy.

**FIGURE 2:**
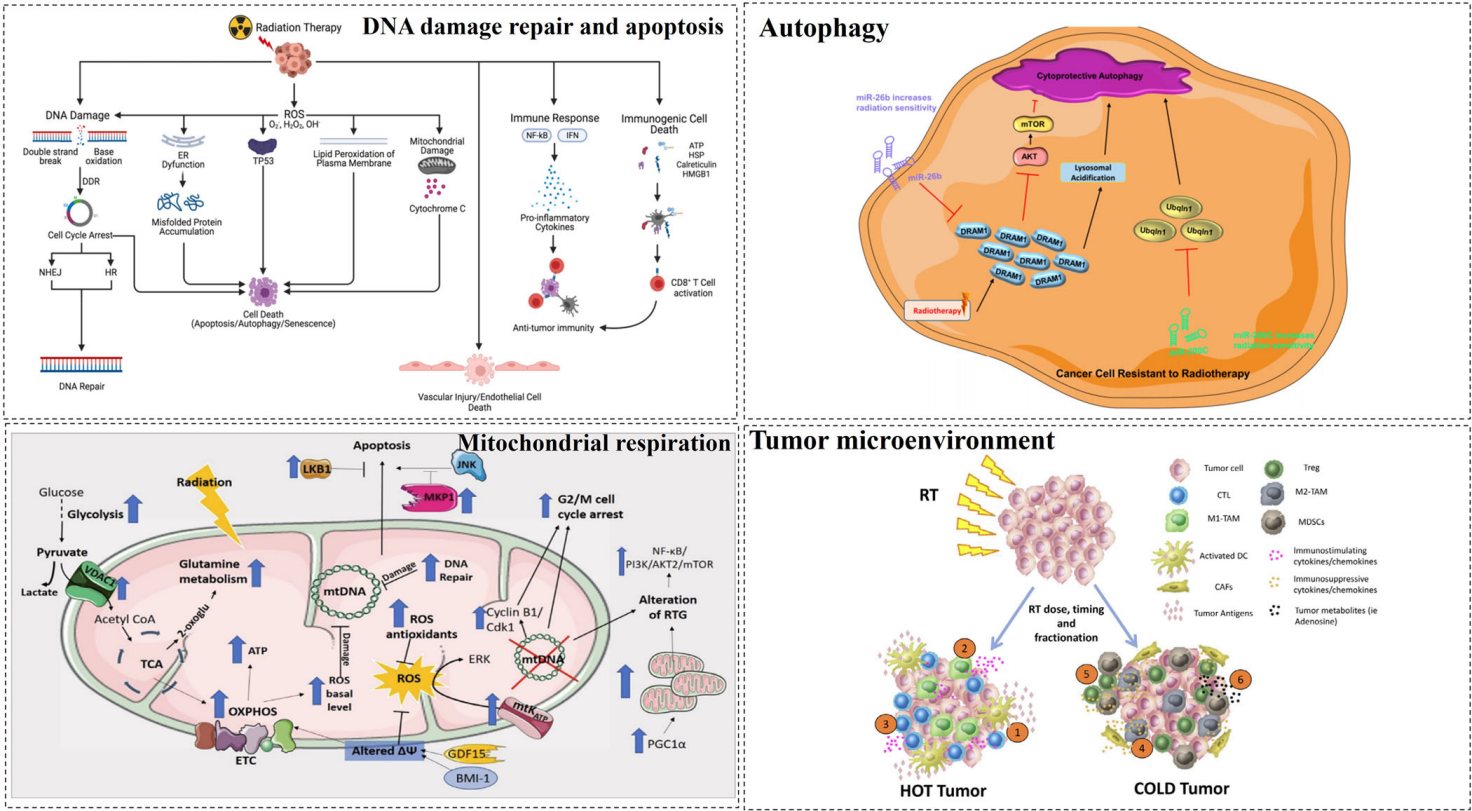
The important molecular mechanisms and signaling pathways of the sensitivity and resistance of radiotherapy in cancer. In the review, we mainly summarized four mechanisms including DNA damage repair, apoptosis, autophagy, mitochondrial respiration, and tumor microenvironment. Reproduced with permission from Ref. [9], © Published by Elsevier Ltd. 2022. Ref. [49], CC BY 4.0 no copyright need. Ref. [78], © Published by Elsevier Ltd. 2021. Ref. [83] © Published by Elsevier Ltd. 2019.

In this part, we have discussed the critical molecular mechanisms and signaling pathways that regulate radiotherapy sensitivity and resistance in cancer (Figure 2). Additionally, emerging studies have identified novel mechanisms, including ferroptosis [94], cuproptosis [95], and CSC‐related determinants [96], as key contributors to radiotherapy response. In addition, cross‐talk between these mechanisms also effect the radiotherapy. For instance, DNA damage plays a crucial role in this process of TME. For example, an interplay between TME and DNA repair variants can result in a multifactorial nature of high‐grade serous ovarian cancer resistance to platinum chemotherapy. In breast cancer, cuproptosis‐related proteins can affect immune cell infiltration, and response to breast cancer immunotherapy through TME. Future research should focus on developing strategies with enhanced efficacy, reduced toxicity, and improved safety profiles to translate these findings into clinical applications.

## 3 Clinical Progress of Enhanced Sensitivity of Radiotherapy in Cancer

Recent advancements in radiotherapy techniques, such as image‐guided radiotherapy, magnetic resonance‐guided adaptive radiotherapy, and proton beam therapy, have significantly improved the precision and accuracy of radiotherapy delivery [97]. However, radiosensitivity is also influenced by tumor biology [98].

**FIGURE 3:**
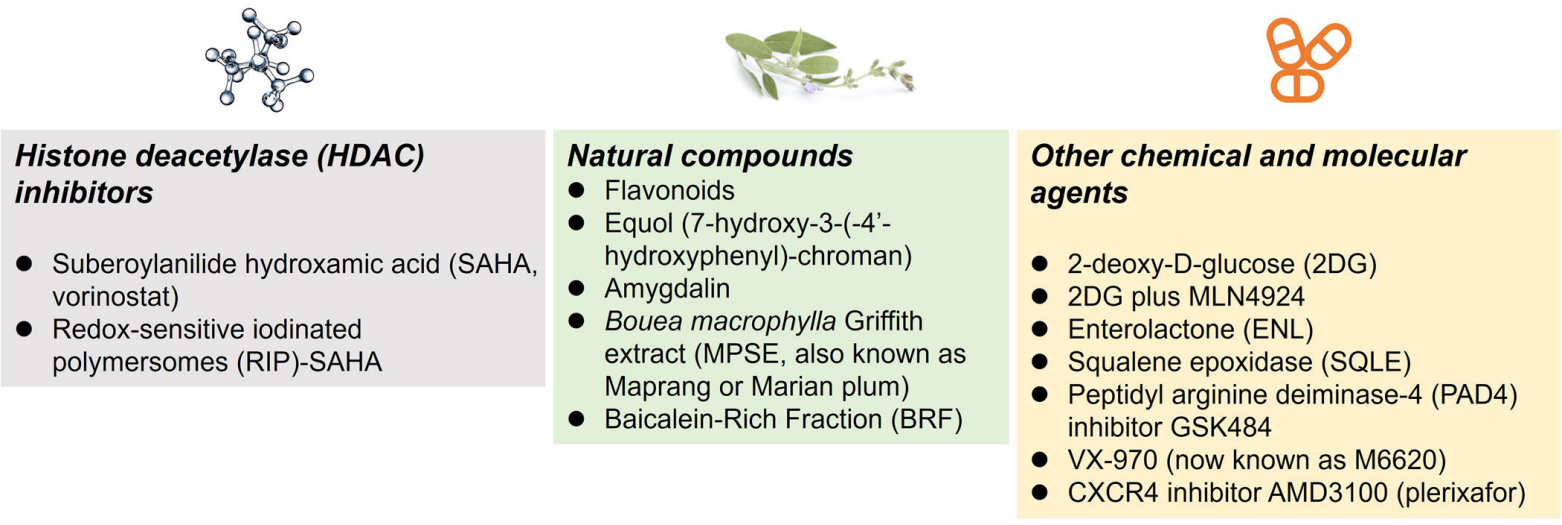
The clinical progress of drugs to affect sensitivity of radiotherapy in cancer. The clinical agents including HDAC inhibitors, natural compounds, and other chemical and molecular agents of enhanced sensitivity of radiotherapy in cancer.

**FIGURE 4:**
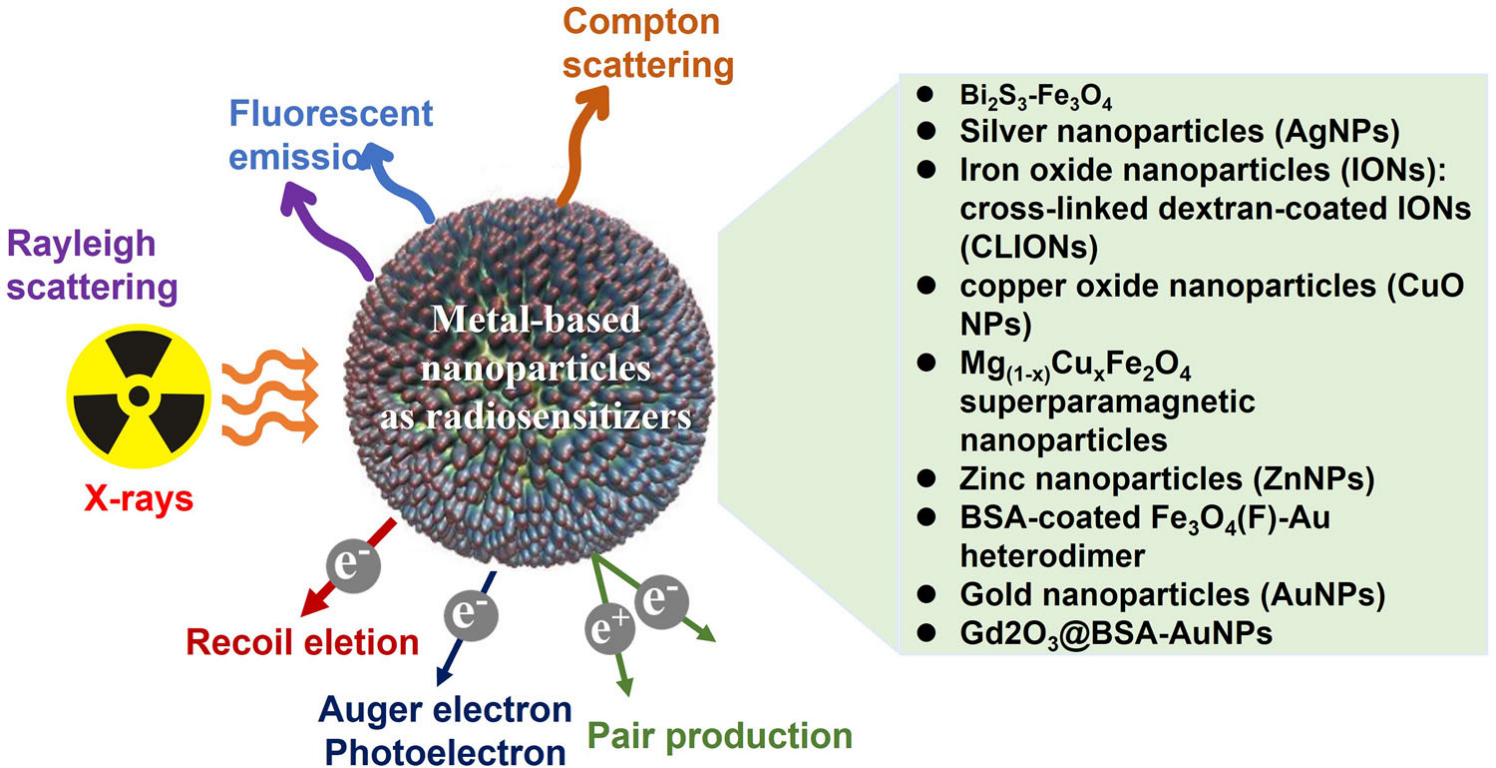
The clinical progress of nanomaterials in radiotherapy. The developing and optimizing various types of metal‐based nanoparticles for radiation‐enhancing purposes in cancer.

For decades, efforts have been made to enhance cancer cell sensitivity to radiotherapy using radiosensitizers. To date, a wide variety of drugs have been identified as potent radiosensitizers [99, 100]. Additionally, natural compounds offer advantages as radiosensitizers due to their minimal or no side effects [101, 102]. This section focuses on clinical advancements in enhancing radiotherapy sensitivity and predicting biomarkers for radiotherapy response in cancer (Figures 3 and 4).

### 3.1 Drugs

#### 3.1.1 Histone Deacetylase Inhibitors

Histone deacetylases (HDACs) play a critical role in regulating transcription by stabilizing DNA–histone interactions and facilitating deacetylation [103, 104]. HDACs contribute to carcinogenesis by influencing cell cycle progression, mitosis, and apoptosis‐related gene transcription [105]. Based on these mechanisms, HDAC inhibitors (HDACi) have been developed to disrupt DNA–histone interactions, increasing DNA exposure and ultimately enhancing radiosensitivity [106].

HDACi have been shown to enhance radiosensitivity in various cancers in both in vitro and in vivo studies. For example, suberoylanilide hydroxamic acid (SAHA; vorinostat) was the first United States Food and Drug Administration‐approved HDACi, initially used for treating cutaneous T‐cell lymphoma and currently undergoing clinical trials for multiple cancer types [107, 108, 109]. However, SAHA has notable side effects, including cardiac toxicity, allergic reactions, and poor tumor‐targeting ability [110]. Additionally, resistance to HDACi often limits their therapeutic success [111].

In breast cancer, Cho et al. [112] investigated the combined effects of the HDACi sodium butyrate and the demethylating agent 5‐Aza‐2′‐deoxycytidine (5‐aza‐DC) on radiosensitivity in RKO colorectal cancer and MCF‐7 breast cancer cell lines. Their results demonstrated that the combination significantly enhanced radiosensitivity in both MCF‐7 and RKO cells [112]. Recently, Zhu et al. [106] developed redox‐sensitive iodinated polymersomes (RIP) carrying SAHA, which significantly enhanced radiotherapy sensitivity in breast cancer. RIP–SAHA, combined with a 4 Gy dose of X‐ray radiation, resulted in significantly increased suppression of breast cancer 4T1 cells. However, neither of these radiosensitizers has been implemented in clinical practice. Combinations of treatments have also been explored to enhance HDACi‐mediated radiosensitization. For pancreatic cancer, HDACi such as TSA and ailibinin, when combined with chemotherapy, demonstrated strong antitumor activity [113, 114]. HDAC6, which modifies non‐histone substrates such as heat shock protein 90, α‐tubulin, and heat shock transcription factor‐1, plays a crucial role in cancer regulation. Small‐molecule inhibitors targeting HDAC6 have been proposed to enhance radiosensitization, driving increased interest in HDACi for improving tumor responses to radiotherapy [115].

More than 18 mammalian HDAC enzymes have been identified, and HDACi represent an emerging class of promising anticancer agents. Some studies suggest that HDACi not only act as radiosensitizers but also reduce radiation‐induced inflammation, such as dermatitis and esophagitis [116].

#### 3.1.2 Other Chemical and Molecular Agents

Among chemotherapy drugs, 2‐deoxy‐d‐glucose (2DG) is a potent inhibitor of glycolysis, glucose metabolism, and ATP production [117]. By depleting cellular energy, 2DG disrupts vital functions and induces apoptosis in cancer cells. Additionally, 2DG suppresses cell growth, reduces colony formation, and enhances apoptosis in cancer cell lines. Oladghaffaria et al. [118] evaluated the combination of 2DG with MLN4924, a novel small‐molecule inhibitor, for sensitizing breast cancer cells to radiation. Their results demonstrated that the combination treatment acted as a potent radiosensitizer, with sensitivity enhancement ratios (SER) of 1.41 and 1.27 in breast cancer SKBR3 and MCF‐7 cells, respectively [118]. Furthermore, 2DG exhibited radioprotective effects by preventing fibrosis development [119].

In addition to chemical agents, certain molecular elements have been identified as novel radiosensitizers. For instance, enterolactone (ENL), a phytoestrogen with weak estrogenic properties, is a dietary lignin structurally similar to endogenous estrogens [120, 121]. Bigdeli et al. [122] conducted cellular experiments demonstrating that ENL enhanced cancer cell radiosensitivity by disrupting X‐ray‐induced G2/M arrest, impairing DNA repair, and increasing apoptosis. Notably, the radiosensitizing effects of ENL were more pronounced in ER‐positive (ER⁺) human breast cancer cell lines (T47D) compared with ER‐negative (MDA‐MB‐231) cells.

Squalene epoxidase (SQLE), an enzyme regulating cholesterol biosynthesis, converts squalene to 2,3‐oxidosqualene [123, 124]. Hong et al. [125] found that SQLE expression was associated with poor prognosis in breast cancer and NSCLC. Inhibiting SQLE enhanced radiosensitivity by regulating the WIP1–ATM axis [125]. Existing SQLE inhibitors, such as terbinafine, may provide a cost‐effective approach for improving radiotherapy outcomes in cancer patients. Wei et al. [126] identified the peptidyl arginine deiminase‐4 inhibitor GSK484 as a potential radiosensitizer. GSK484 enhanced IR‐induced suppression of cell proliferation, migration, and invasion, suggesting its role in sensitizing TNBC cells by inhibiting IR‐induced autophagy [126].

In TNBC, Tu et al. [127] demonstrated that the ataxia telangiectasia mutated and Rad3‐related protein kinase (ATR) inhibitor VX‐970 (M6620) preferentially sensitized TNBC cells to radiotherapy by abrogating radiation‐induced cell cycle checkpoints and inhibiting DNA DSB repair in vivo. Similarly, Zhou et al. [128] performed in vivo and in vitro analyses, revealing that the CXCR4 inhibitor AMD3100 (plerixafor) sensitized TNBC cells to radiation. Additionally, ribophorin II, a component of the N‐oligosaccharyl transferase complex, plays a role in apoptosis regulation. Its silencing has been shown to confer tumor sensitivity to cisplatin‐based treatments [129].

#### 3.1.3 Natural Compounds

Natural products and compounds are increasingly recognized as promising radiosensitizers due to their minimal side effects and inherent radiosensitizing properties [102, 130]. Several studies have demonstrated that natural compounds enhance tumor cell sensitivity to radiation by impairing DNA repair mechanisms.

Among these, flavonoids, a class of natural plant‐derived compounds, offer numerous health benefits, including cancer treatment and radioprotection of normal cells. Additionally, flavonoids can sensitize tumor cells to radiation [102]. They are secondary metabolites widely distributed in plants, consisting of two benzene rings (A and B) linked via a heterocyclic pyrone ring (C) [131, 132]. Flavonoids are categorized into six subclasses: flavones, flavanones, flavanols, flavonols, isoflavones, and anthocyanidins. Puthli et al. [133] reported that the flavonoid biochanin A increased ROS production when combined with radiation in colon tumor cells. Previous studies have shown that quercetin, a naturally occurring flavonoid, enhanced radiosensitivity in vitro in MCF‐7 breast cancer cells, with a SER of 1.74. Furthermore, pretreatment with 10 mM genistein for 24 h, followed by 4 Gy radiation, increased radiosensitivity in MCF‐7 and MDA‐MB‐231 breast cancer cells [134]. Flavonoids also demonstrate efficacy against radiation‐resistant breast cancer by inducing apoptosis [135] and inhibiting tumor growth [136]. Additionally, the flavonoid fisetin has been shown to inhibit RSK‐mediated YB‐1 signaling, thereby increasing radiosensitivity in colorectal cancer cells [137]. Similar findings suggest that flavonoid compounds enhance radiosensitivity in various tumor cells [138, 139].

Beyond flavonoids, Equol (7‐hydroxy‐3‐(4′‐hydroxyphenyl)‐chroman), a metabolite of the soy isoflavone daidzein, has demonstrated anticancer effects. Approximately 30–50% of adults can metabolize daidzein into equol, suggesting its potential therapeutic effectiveness [140, 141]. Taghizadeh et al. [142] found that pretreatment with 50 µM equol for 72 h in MDA‐MB‐231 cells and 24 h in T47D cells increased radiosensitivity, accompanied by increased radiation‐induced DNA damage. Amygdalin, a naturally occurring vitamin B17, has also received attention for its anticancer properties. Found in seeds of Prunus Rosaceae family plants (e.g., apricots, apples, bitter almonds, black cherries, plums, peaches), amygdalin induces apoptosis and cell cycle arrest by hydrolyzing into hydrogen cyanide, selectively killing cancer cells [143, 144]. Askar et al. [145] developed amygdalin‐folic acid nanoparticles, which inhibited cancer proliferation and enhanced radiotherapy effects in vitro.

Other natural products, such as Bouea macrophylla Griffith extract (MPSE), commonly known as Maprang or Marian plum, have also shown anticancer effects. Kantapan et al. [146, 147] demonstrated that MPSE pretreatment reduced radiation‐induced activation of prosurvival pathways by decreasing ERK and AKT phosphorylation, thereby sensitizing cancer cells to radiation in vitro. Similarly, Baicalein‐rich fractions have been proposed as non‐toxic radiosensitizers that enhance radiotherapy sensitivity in breast cancer cells [148]. Huaier, a traditional medicinal fungus, has also demonstrated significant radiosensitization effects in breast cancer through cell cycle regulation and DNA repair pathway modulation [149].

To overcome challenges such as low aqueous solubility, bioavailability, and tumor specificity, recent studies have explored combining natural products with nanoformulations to improve their therapeutic potential [150]. However, most findings on the radiosensitization effects of natural compounds are based on cellular experiments. Future clinical studies are needed to evaluate their toxicity and efficacy in cancer treatment.

### 3.2 Technologies

#### 3.2.1 Metal‐Based Nanoparticles

Metal‐based nanoparticles have gained significant attention as radiosensitizers due to their ability to enhance X‐ray absorption. Specifically, materials containing high atomic number (Z) elements, such as gold (gold nanoparticles [AuNPs]), gadolinium‐based, and platinum‐based nanoparticles, improve radiotherapy efficacy [151, 152, 153, 154, 155]. For example, in breast cancer treatment, Nosrati et al. [156] developed a bimetallic nanoradiosensitizer (Bi₂S₃–Fe₃O₄), which promoted radiation‐induced DNA damage while reducing adverse effects in a 4T1 breast cancer murine model upon X‐ray exposure. Lu et al. [157] synthesized silver nanoparticles using egg white and found that they enhanced X‐ray IR sensitivity in MDA‐MB‐231 breast cancer cells. Similar findings were observed in TNBC cells in vitro and in vivo [158].

Iron oxide nanoparticles (IONs) have also been investigated for their radiosensitization potential. Huang et al. [159] demonstrated that cross‐linked dextran‐coated IONs (CLIONs) improved X‐ray IR cytotoxicity in EMT‐6 murine breast cancer cells. Additionally, Jiang et al. [160] explored the use of copper oxide nanoparticles (CuO NPs) as nanoradiosensitizers. Their study showed that autophagy‐inducing CuO NPs significantly enhanced radiosensitivity in MCF‐7 breast cancer cells, particularly when combined with X‐ray IR. While CuO NPs alone did not inhibit tumor growth, their combination with X‐ray radiation resulted in a prolonged antitumor effect compared with radiation alone. Similarly, Meidanchi et al. [161] developed Mg(1–*x*)CuxFe₂O₄ superparamagnetic nanoparticles, which significantly enhanced radiotherapy sensitivity in MCF‐7 cells without inducing cytotoxicity.

Beyond pure metal nanoparticles, metal–drug hybrid nanoparticles have also been developed as radiosensitizers. Due to their selective uptake by malignant cells and unique physicochemical properties, zinc nanoparticles have been explored as multifunctional cancer treatment agents. Asadi et al. [162] synthesized Zn@Alg–Dox NPs, which were coated with doxorubicin‐conjugated alginate. These nanoparticles effectively functioned as radiation sensitizers in TNBC cells (MDA‐MB‐231). Nosrati et al. [151] designed BSA‐coated Fe₃O₄–Au heterodimer nanostructures, which functioned as radiosensitizers while codelivering chemotherapeutic drugs, enhancing radiotherapy in breast cancer models. Igaz et al. [163] examined the combination of AuNPs with the HDACi SAHA, finding that the AuNP + SAHA combination significantly increased radiosensitivity in multiple tumor cell lines, including MCF‐7 breast cancer cells.

Interestingly, Tudda et al. [164] demonstrated that AuNPs exhibited strong radio‐enhancing effects when combined with 190 kV photons in MDA‐MB‐231 breast cancer cells. More recently, Nosrati et al. [165] developed bovine serum albumin‐capped gadolinium oxide and gold nanoparticles (Gd₂O₃@BSA–AuNPs) as bimetallic radiosensitizers. Their study showed significant improvement in cancer therapy efficacy in a breast cancer murine model under X‐ray IR, with no observed toxicity to healthy tissues [165]. Moreover, exosome‐based nanozymes have emerged as promising candidates for radiotherapy enhancement. Chen et al. synthesized a tumor cell exosome‐mimicking multifunctional nanozyme, which demonstrated significant radiosensitization effects in breast cancer in vitro and in vivo [166, 167, 168].

While numerous studies have optimized metal‐based nanoparticles for radiation enhancement, most have yet to be validated in clinical trials [169, 170]. Future research should focus on translating these findings into clinical applications through well‐designed trials evaluating safety and efficacy.

#### 3.2.2 Gene Editing

Beyond drugs and molecular factors, the heterogeneity of patient genomic expression has been linked to radiotherapy sensitivity in cancer. This phenomenon is referred to as intrinsic radiosensitivity, which describes inherent differences in tumor cell sensitivity to radiation, independent of biological factors such as hypoxia or proliferation [171, 172].

Among various genetic factors, p53 has been extensively studied as a potential target for radiosensitization and radioprotection [173]. Mutations and epigenetic modifications of p53 influence radiosensitivity in solid tumors, although the underlying mechanisms remain unclear. For instance, although the first clinically applicable HDACi, benzamide MS‐275, has demonstrated radiosensitizing potential, its mechanism of action via p53 modulation is yet to be elucidated [174, 175]. Similar uncertainties exist in other studies investigating genetic influences on radiosensitivity.

Focusing on breast cancer, Chen et al. [176] employed deep learning models to identify a radiotherapy sensitivity signature comprising six key genes (HOXB13, NKX2‐2, ADAMTS20, LOC284930, ACTL8, and LOC101928978). Additionally, Zhang et al. [177] found that the expression of fragile‐site‐associated tumor suppressor correlated with radiotherapy sensitivity in breast cancer. Furthermore, Hu et al. [178] reported that patients with high levels of Holliday Junction Recognition Protein exhibited significantly increased sensitivity to radiotherapy. However, the precise mechanisms by which these genetic mutations impact radiosensitivity remain unclear [179].

Additionally, miRNAs and circular RNAs (circRNAs) have been implicated in regulating radiotherapy sensitivity. For instance, in vitro studies on breast cancer cell lines (MDA‐MB‐231 and SKBR3) revealed that miR‐144 overexpression significantly increased radiation resistance [179]. Conversely, Jiang et al. [180] found that miR‐4796 enhanced radiation‐induced cell death by directly targeting multiple DDR components, thereby repressing DNA repair pathways. Moreover, circRNAs such as Circ‐HIPK3 and Circ‐PVT1 have been linked to radiosensitivity in cancer patients [181]. Similarly, Wang et al. [182] reported that the long noncoding RNA LINC02582, acting downstream of miR‐200c, promoted radioresistance by regulating CHK1 signaling.

These findings suggest that genetic and noncoding RNA markers could help physicians identify radiosensitive patients more efficiently before radiation therapy. Moreover, targeting noncoding RNAs may serve as an effective strategy for radiation sensitization.

#### 3.2.3 Kochi Oxydol‐Radiation Therapy for Unresectable Carcinomas II

Most cancer types contain hypoxic tumor cells and high levels of antioxidative enzymes, making them inherently resistant to radiotherapy. To address this challenge, a novel radiosensitizer known as Kochi Oxydol‐Radiation Therapy for Unresectable Carcinomas (KORTUC II) has been developed [183, 184]. Currently, KORTUC II is the most widely used radiosensitizer in Japan [185].

KORTUC II enhances radiosensitivity by converting hypoxic and radioresistant cancer cells into hyperoxic and radiosensitive cells, thereby improving therapeutic outcomes. Recent clinical trials in Japanese populations have demonstrated its efficacy as a hydrogen peroxide‐based radiosensitizer for enhancing brachytherapy in cancers such as cervical and breast cancer [186, 187, 188]. However, clinical trials in non‐Japanese populations have yet to confirm its efficacy, warranting further international studies.

### 3.3 Clinical Trial Progress

While numerous preclinical studies have highlighted the potential of various radiosensitizers in cancer treatment, their precise effects on radiosensitivity remain unclear, and only a limited number of clinical trials have validated their efficacy.

Clinical research on chemical radiosensitizers began in the 1970s. The first agent, metronidazole, was introduced in 1974, but only a few studies demonstrated significant benefits [189]. Subsequent research identified the antilipidemic drug efaproxiral (RSR13) as a radiosensitizer [190]. Multiple clinical trials have evaluated efaproxiral in patients undergoing radiotherapy for tumor metastases and locally advanced unresectable cancers, with some positive findings. Additionally, cetuximab and alpelisib (BYL719) have been investigated as radiosensitizing agents for treating head and neck squamous cell carcinoma [191]. Another agent, nelfinavir, was found to enhance intrinsic radiosensitivity, and when combined with chemoradiotherapy, it showed acceptable toxicity and promising survival outcomes in pancreatic cancer [192]. Moreover, pyrotinib treatment improved radiosensitivity in HER2‐positive brain metastatic breast cancer [193]. (Table 1)

**TABLE 1:** The Clinical Trial Progress of radiosensitizers in cancer treatment.

Category	Name	The key information of clinical trials
Drugs	Metronidazole [189]	Misonidazole was first given in clinic in 1974. However, the results so far recorded of the clinical trials with misonidazole have been generally disappointing. Only in five out of 32 studies analyzed have significant benefits been shown to suggest real advantage with the use of misonidazole.
	Efaproxiral (RSR13) [190]	Multiple clinical trials have evaluated efaproxiral (RSR13) in patients undergoing radiotherapy for tumor metastases and locally advanced unresectable cancers. Phase I–III trial data have defined the safety profile and dosing of the drug, with the potential benefit for extended survival.
	Cetuximab, alpelisib (BYL719) [191]	Etuximab and alpelisib (BYL719) have been investigated as radiosensitizing agents for treating head and neck squamous cell carcinoma (HNSCC). BYL719 likely enhances common toxicities associated with definitive radiation and cetuximab.
	Nelfinavir [192]	Chemoradiotherapy combined with nelfinavir showed acceptable toxicity and promising survival in pancreatic cancer. In addition, the toxicity was manageable.
	Pyrotinib [193]	Oral admission of pyrotinib together with radiotherapy can significantly increase the overall response rate, progression‐free survival, time to progression and duration of response of HER2^+^ brain metastatic breast cancer patients, without causing extra adverse events. Pyrotinib might be an effective medication to enhance the tumor radiosensitivity of HER2‐positive brain metastatic breast cancer patients.
Genetic factors	Stromal PDGFRβ expression [194]	High stromal PDGFRb expression as a novel biomarker identifying ductal carcinoma in situ (DCIS) patients who are refractory to standard whole‐breast adjuvant radiotherapy
	Ku70 expression [195]	Treatment with 76 Gy and ADT can be effective for patients with Gleason score ≤ 7 or low Ku70 expression, but is not enough for patients with Gleason score ≥ 8 and high Ku70 expression and require other treatment approaches.
	miR‐145 [196]	MiR‐145 overexpression enhancing radiation response in vivo

Genetic factors have also been explored in clinical trials. For example, high stromal PDGFRβ expression was identified as a novel biomarker for predicting radiosensitivity in ductal carcinoma in situ (DCIS) [194]. Similarly, Ku70 expression was found to correlate with radiotherapy outcomes in prostate cancer [195]. Regarding noncoding RNAs, miR‐145 was shown to significantly modulate tumor sensitivity to radiation in prostate cancer [196]. A notable clinical trial by Skiöld et al. [197] investigated radiation sensitivity mechanisms, providing insights essential for personalized radiation therapy. However, compared with chemical agents and genetic biomarkers, metal‐based nanoparticle radiosensitizers have yet to demonstrate efficacy in clinical settings. Future clinical trials are necessary to translate these findings into clinical practice (Table 1).

In recent years, radiation oncologists have identified key factors influencing radiotherapy sensitivity and developed predictive biomarkers to determine which cancer patients would benefit most from radiotherapy [18, 198]. Radiosensitizing agents, including HDACi, chemical and molecular agents, natural compounds, metal‐based nanoparticles, KORTUC II, and genetic and noncoding RNA markers, have demonstrated potential in enhancing radiotherapy sensitivity, particularly in breast cancer. These predictive markers can assist oncologists in optimizing radiotherapy dosing while balancing anticancer efficacy and minimizing toxicity. However, only a few clinical trials have been conducted to confirm their effectiveness. Future research should prioritize clinical and translational studies to validate radiosensitization targets and integrate them into standard oncologic practice.

## 4 Side‐Effects of Radiotherapy in the Treatment of Cancer: Take Breast Cancer as an Example

**FIGURE 5:**
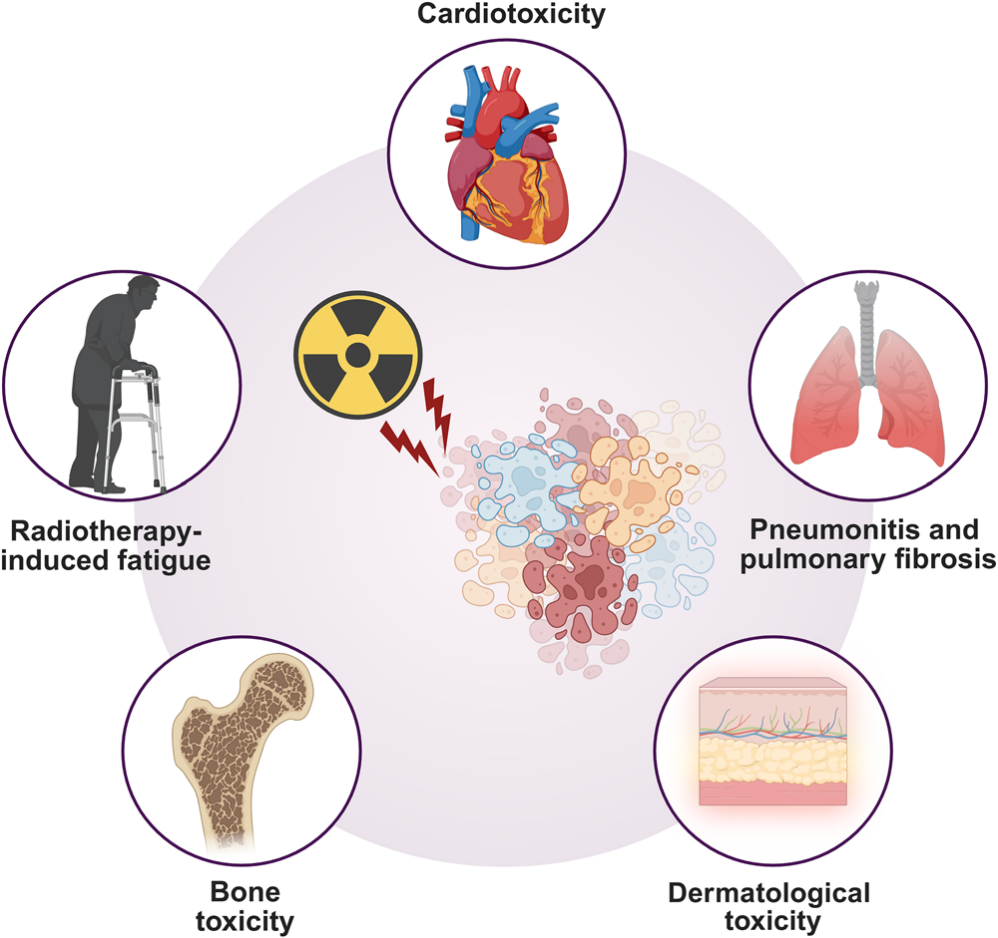
The main damaged organs suffered from radiotherapy. Besides the heart effect, pneumonitis, and pulmonary fibrosis, radiotherapy‐induced fatigue, osteoporosis, and progressive hyalinization and fibrosis of medullary spaces are also observed in breast cancer patients undergoing radiotherapy. In addition, the other side effects can be observed in the skin, ovary, and uteri. This figure was created using BioRender.

It is impossible to ignore that many patients face early and late side effects following radiotherapy. There has been increasing concern that radiation‐induced heart disease (RIHD) offset the benefits of radiotherapy. That is because with longer periods of survival, patients with cancers are willing to gain a higher life quality. Recently, the early and late effects of chest radiation are the main challenge faced by most clinicians. Among them, RIHD is a major concern. Additionally, several other complications remain unavoidable despite advances in treatment strategies. In addition, attention should be paid about the side effects of radiotherapy and immune checkpoint inhibitors combination strategy. Furthermore, rapidly dividing cells such as gastrointestinal cells are susceptible to the effects of radiotherapy. Radiation can also deplete bone marrow resulting in cytopenias. In addition, late toxicities from radiotherapy include bowel obstruction, ulceration, perforation, and bleeding. In this section, it reviews the most common and clinically significant complications associated with radiotherapy in breast cancer as an example (Figure 5).

### 4.1 Cardiotoxicity

Radiotherapy can cause secondary cardiac damage, particularly when the heart is within the radiation field [199, 200, 201]. For example, studies have shown that breast cancer survivors undergoing radiotherapy face a 1.7‐fold increased risk of cardiovascular mortality, with risk increasing over time [199, 202]. Darby et al. [203] reported a 7.4% increase in ischemic heart disease per one Gray (Gy) increase in radiation dose absorbed by the heart.

Direct ionization leads to damage in cardiac cellular components and induces water radiolysis, which can cause coronary artery disease, cardiomyopathy, pericarditis, acute myocardial infarction, valvular disorders, fibrosis, and heart failure [203, 204, 205]. The extent of cardiotoxic effects depends on the radiation dose, tumor location, and the volume of heart tissue exposed [206].

Interestingly, differences in cardiac toxicity between right‐ and left‐sided breast cancer have been observed, likely due to the use of intensity‐modulated radiotherapy, which is more commonly applied for left‐sided tumors [207, 208]. To mitigate cardiotoxicity, conformal and intensity‐modulated techniques have been introduced to minimize heart exposure during adjuvant breast cancer treatment [209, 210].

Recent studies have also highlighted nanomedicine approaches, such as micelle‐based drug delivery, which show potential in treating radiation‐induced cardiac damage, particularly fibrosis [211]. Furthermore, respiratory motion management and proton therapy have been proposed as strategies to reduce radiation‐induced cardiac toxicity.

### 4.2 Pneumonitis and Pulmonary Fibrosis

Pulmonary toxicity is another major side effect of radiotherapy, ranging from asymptomatic radiological changes to severe respiratory failure [210]. The clinical presentation varies from mild to severe respiratory impairment. Since chest wall radiotherapy is an essential component of breast cancer management after surgery [212], radiation‐induced pneumonitis and pulmonary fibrosis remain unavoidable risks. Radiation‐induced lung injury typically manifests in three forms:

1. Radiation pneumonitis—an early inflammatory response
2. Organizing pneumonia—a subacute reaction
3. Radiation recall pneumonitis—an inflammatory response triggered by certain chemotherapies [213]

Currently, the treatment of drug‐ and radiotherapy‐induced pneumonitis remains empirical and based on symptom severity. Mehnati et al. [214] proposed a predictive model using pulmonary function parameters, particularly forced expiratory volume in 1 s, to assess the risk of radiation pneumonitis following breast cancer radiotherapy.

### 4.3 Bone Toxicity

Bone toxicity, particularly radiation‐induced rib fractures and osteoradionecrosis, is a recognized complication of radiotherapy. A meta‐analysis of 57 studies including 5,985 cases identified a 2% incidence of rib fractures following conventional fractionated radiotherapy [215]. Most rib fractures are asymptomatic, but their risk correlates with tumor proximity to the rib cage [216]. Key risk factors for radiation‐induced rib fractures include:

- High rib dose‐volume exposure
- Location of the radiation field
- Use of stereotactic body radiation therapy (SBRT) [217]

Pettersson et al. [218] found that hypofractionated SBRT increased rib fracture risk, particularly when the dose to 2 cm^3^ of rib bone exceeded the threshold. To minimize risk, studies suggest limiting chest wall doses exceeding 30 Gy to less than 30 cm^3^ [218].

Another rare but severe complication is osteoradionecrosis, particularly in locally advanced breast cancer patients requiring high radiation doses [219]. Pandey et al. [219] reported that total radiation dose is the primary risk factor for osteoradionecrosis.

Management strategies include:

- Antibiotics
- Hyperbaric oxygen therapy
- Sequestrum removal (surgical excision of necrotic bone)

For severe cases requiring reconstructive surgery, Arya et al. [220] described microsurgical techniques to repair large chest wall defects caused by osteoradionecrosis. Although chest wall osteoradionecrosis is rare, it remains a severe complication, often difficult to differentiate from bone metastases [221].

### 4.4 Dermatological Toxicity

Breast dermatofibrosis is a common complication in breast cancer patients following local breast radiotherapy [222]. Breast fibrosis is often associated with poor cosmetic outcomes, leading to psychosocial distress in some patients [223]. To mitigate and assess the risk of dermatofibrosis, several predictive tests have been developed for personalized radiation therapy. For example, Azria et al. [224] conducted a prospective multicenter clinical trial, which revealed a significant inverse correlation between radiation‐induced lymphocyte apoptosis (RILA) in CD8⁺ T cells and radiation‐induced fibrosis. In this study, 456 breast cancer patients were followed for up to 36 months, and the findings demonstrated that RILA was a strong predictor of breast fibrosis. Similarly, Pereira et al. [225] investigated nuclear autophosphorylated ATM protein (pATM) as a predictor of clinical radiosensitivity using an enzyme‐linked immunosorbent assay. Their results suggested that measuring nuclear pATM levels after a 2 Gy radiation dose could serve as a highly reliable predictive biomarker for fibrosis risk. Furthermore, a recent frequency‐matched study by McMahon et al. [226] analyzed preradiotherapy urine samples from 60 breast cancer patients using metabolomics. The results identified key metabolic pathways, including alanine, aspartate, and glutamate metabolism, as the most impacted by radiotherapy‐induced adverse skin reactions. Additionally, C‐reactive protein, an inflammatory biomarker, was also found to be associated with early radiation‐induced skin toxicity in breast cancer patients [227]. These findings highlight the importance of biomarker‐based assessment to predict and potentially mitigate dermatological toxicity in radiotherapy.

### 4.5 Radiotherapy‐Induced Fatigue

Radiotherapy‐induced fatigue (RIF) is one of the most common side effects of radiotherapy in breast cancer patients [228, 229]. Studies suggest that over 70% of breast cancer patients undergoing radiotherapy experience fatigue‐related disorders, significantly affecting daily activities [230]. Risk factors for RIF include smoking, younger age, and long travel distances to treatment centers [231].

Several mechanisms contribute to RIF, including:

1. *Oxidative stress and mitochondrial dysfunction*—Ionizing radiation generates ROS, leading to oxidative damage in late‐responding healthy tissues. This mutates mitochondrial DNA, further amplifying ROS production and fatigue [232].
2. *Anemia and functional iron deficiency*—These conditions exacerbate fatigue by impairing oxygen transport and energy metabolism [233, 234].

*Management strategies*: Current clinical guidelines recommend multidisciplinary interventions, including:

- Physical therapy
- Occupational therapy
- Psychosocial interventions [235, 236]

Despite its prevalence, RIF is often underreported, underdiagnosed, and undertreated. Additionally, standardized evaluation tools for assessing RIF remain lacking, highlighting the need for improved clinical assessment protocols.

## 5 Challenges, Conclusions, and Prospects

As we know, intrinsic or acquired tumor radioresistance significantly affects the effectiveness of cancer treatment. Recently, radiosensitizers have faced challenges in clinical translation, not only due to complex manufacturing techniques but also high costs. In clinical applications, radiotherapy is dose‐limited due to its potential toxicity to normal tissues. Therefore, for radiosensitizers to be clinically useful, they must enhance the efficacy of radiotherapy while increasing the probability of tumor cure without causing additional toxicity. Kareliotis et al. analyzed various current cancer therapy options and concluded that multimodal treatment, combining ionizing radiation and sensitizing agents, is essential [237, 238]. However, increasing radiation doses or sensitization using chemotherapeutic agents often leads to higher toxicity and morbidity. Moreover, most radiosensitizing agents reviewed in research have not demonstrated clinical efficacy. More clinical trials are necessary to advance their real‐world applications.

In conclusion, future research should focus on identifying potential radiosensitization targets, developing new radiosensitizing strategies, and discovering molecular biomarkers for accurate prediction and assessment of radiosensitivity in cancer. Most previous studies were conducted at the cellular or animal level, with only a few translated into clinical applications. The success rate of transitioning radiosensitization research into clinical practice remains low, particularly regarding radiosensitization targets [239].

Here, we propose solutions to key challenges. First of all, many challenges persist in enhancing radiotherapy sensitivity, including biological and clinical factors. Tumor heterogeneity and adaptive resistance mechanisms limit radiosensitivity. Additionally, patient variability in radiotherapy response also influences radiosensitivity.

Second, while radiotherapy is effective in reducing locoregional recurrence, there is still no clinical tool to determine which patients may safely forgo adjuvant radiation therapy [240]. Recent studies identified risk factors such as older age and smaller tumor size as predictors of low locoregional recurrence risk [241]. For example, Cui et al. [242] established a radiosensitivity signature incorporating age, ER status, HER2 status, tumor stage, hormone therapy, chemotherapy, and surgery, to identify patients most likely to benefit from radiotherapy. Additionally, Sjöström et al. [243] introduced the Adjuvant Radiotherapy Intensification Classifier, a 27‐gene signature combined with patient age, to predict radiotherapy benefit in breast cancer. Similarly, Tramm et al. [244] identified a seven‐gene classifier predicting radiotherapy benefit in high‐risk breast cancer patients treated with systemic therapy. However, while biomarkers are commonly used to predict chemotherapy response, validated genomic markers for radiotherapy efficacy remain scarce [245, 246, 247, 248, 249]. With the rise of precision medicine, multiomics approaches are improving our understanding of cancer biology [250, 251, 252]. Recent proteo‐transcriptomic studies have uncovered novel biological mechanisms of intraoperative radiotherapy‐treated tumors, suggesting the need for personalized postoperative radiotherapy decisions [253]. Future treatment strategies should integrate clinical and genomic data to optimize radiotherapy planning.

Third, certain cancers frequently metastasize to the brain, where current therapies such as stereotactic radiosurgery and whole‐brain radiotherapy (WBRT) have limited efficacy due to the blood–brain barrier (BBB) [254]. Additionally, brain metastases and their treatments significantly affect neurocognitive function and quality of life, especially in WBRT patients. For instance, breast cancer brain metastases (BCBM) are associated with:

- Younger age
- Larger tumor size
- Higher tumor grade
- Positive lymph node involvement
- Expression of Ki‐67 (a proliferation marker) [255]

Among breast cancer subtypes, TNBC and HER2‐positive breast cancer exhibit higher rates of brain metastases [256]. Once tumor cells infiltrate the brain, they trigger neo‐angiogenesis and microenvironment remodeling, fostering tumor progression and invasion [257, 258]. Unlike the intact BBB, the blood–tumor barrier has unique molecular properties that complicate treatment strategies.

Despite advances in early detection and therapy, the 5‐year survival rate for BCBM remains below 30% [259]. As brain metastases develop multidrug resistance, the TME supports metastatic progression, presenting further treatment challenges [260, 261]. To enhance radiotherapy for brain metastases, researchers are exploring:

- Intrathecal and intraarterial drug delivery
- Nanoparticle‐based radiosensitizers

For example, Gd‐based AGuIX nanoparticles have been shown to enhance radiotherapy efficacy and extend survival in both in vitro and in vivo models [262, 263]. A recent human trial involving 15 patients with four types of brain metastases, including breast cancer, demonstrated that MRI signal enhancement correlated with tumor response, suggesting that nanoparticles could improve radiotherapy effectiveness [264]. Additionally, combining radiotherapy with targeted therapies has shown promising results. Patients with glioblastoma treated with radiotherapy plus temozolomide achieved longer survival compared with radiotherapy alone [265]. Likewise, cilengitide, an integrin inhibitor, was shown to enhance radiation response in preclinical breast cancer models [266].

Another approach is radio‐immunotherapy, which has improved tumor control and survival rates in experimental breast‐to‐brain metastasis models [267, 268]. Recent studies by Niesel et al. [269] found that radiotherapy can enhance immunogenicity, increasing the effectiveness of immune checkpoint inhibitors. Despite these advancements, clinical strategies to improve radiotherapy for brain metastases remain limited. Future research should focus on:

1. Minimizing neurocognitive decline from WBRT
2. Enhancing radiosensitizers to penetrate the BBB
3. Optimizing combination therapies for tumor control
4. Expanding clinical trials for new radiosensitizers

Furthermore, collaboration among oncologists, radiologists, and neuroscientists is crucial for translating preclinical findings into clinical applications more efficiently. The toxicity of a substance depends on dosage and individual sensitivity. At high doses, any substance can be toxic. Radiosensitizers can significantly reduce radiation doses while maintaining anticancer efficacy. Additionally, a more demographically centered approach to radiation toxicity management could facilitate individualized interventions and supportive care [269]. Similarly, Jandu et al. conducted a genome‐wide association study on treatment‐related toxicity 2 years after radiotherapy and found significant associations between specific SNPs and toxicity endpoints [270, 271]. Based on these findings, we propose that developing “gene and protein information cards” for cancer patients could enhance personalized cancer treatment and management in the future [272].

In summary, recent advances in radiotherapy technology have significantly optimized treatment outcomes. Together with improved radiosensitizers, radiotherapy has become more effective. However, the application of radiosensitizers in radiotherapy still presents challenges. Compared with chemical drugs, the combination of nanoparticles with radiotherapy sensitizers has shown great potential in improving treatment efficacy. Among these approaches, multifunctional nanoparticles have shown greater clinical applicability than single‐functional nanoparticles. In the future, new drug delivery systems may enhance the radiosensitizing effects of these agents [271, 273, 274]. Furthermore, future research will focus on enhancing the drug‐carrying capacity of nanomaterials to further improve radiotherapy outcomes.

## Author Contributions

YY. Z., FQ. T., and JJ. Z. conducted the study and wrote the manuscript. C. G. and Y. L. designed the research, revised the manuscript, and supervised the study. SC. Z. provided technical support. All authors have read and approved the final version of the manuscript.

## Conflicts of Interest

All authors declare no conflicts of interest.

## Ethics Statement

The authors have nothing to report.

## Data Availability

The authors have nothing to report.
